# Phosphate-Dependent Regulation of Growth and Stresses Management in Plants

**DOI:** 10.3389/fpls.2021.679916

**Published:** 2021-10-28

**Authors:** Noura Bechtaoui, Muhammad Kabir Rabiu, Anas Raklami, Khalid Oufdou, Mohamed Hafidi, Martin Jemo

**Affiliations:** ^1^AgroBiosciences Program, University Mohammed VI Polytechnic (UM6P), Benguerir, Morocco; ^2^Centre for Dryland Agriculture, Bayero University, Kano, Nigeria; ^3^Laboratory of Microbial Biotechnology, Agrosciences, and Environment (BioMAgE), Faculty of Sciences Semlalia, Cadi Ayyad University, Marrakech, Morocco

**Keywords:** adaptation, phosphate, plant growth, stress tolerance, phosphorus

## Abstract

The importance of phosphorus in the regulation of plant growth function is well studied. However, the role of the inorganic phosphate (Pi) molecule in the mitigation of abiotic stresses such as drought, salinity, heavy metal, heat, and acid stresses are poorly understood. We revisited peer-reviewed articles on plant growth characteristics that are phosphorus (P)-dependently regulated under the sufficient-P and low/no-P starvation alone or either combined with one of the mentioned stress. We found that the photosynthesis rate and stomatal conductance decreased under Pi-starved conditions. The total chlorophyll contents were increased in the P-deficient plants, owing to the lack of Pi molecules to sustain the photosynthesis functioning, particularly, the Rubisco and fructose-1,6-bisphosphatase function. The dry biomass of shoots, roots, and P concentrations were significantly reduced under Pi starvation with marketable effects in the cereal than in the legumes. To mitigate P stress, plants activate alternative regulatory pathways, the Pi-dependent glycolysis, and mitochondrial respiration in the cytoplasm. Plants grown under well-Pi supplementation of drought stress exhibited higher dry biomass of shoots than the no-P treated ones. The Pi supply to plants grown under heavy metals stress reduced the metal concentrations in the leaves for the cadmium (Cd) and lead (Pb), but could not prevent them from absorbing heavy metals from soils. To detoxify from heavy metal stress, plants enhance the catalase and ascorbate peroxidase activity that prevents lipid peroxidation in the leaves. The *HvPIP* and *PHO1* genes were over-expressed under both Pi starvation alone and Pi plus drought, or Pi plus salinity stress combination, implying their key roles to mediate the stress mitigations. Agronomy Pi-based interventions to increase Pi at the on-farm levels were discussed. Revisiting the roles of P in growth and its better management in agricultural lands or where P is supplemented as fertilizer could help the plants to survive under abiotic stresses.

## Introduction

The growing population in the world likely will attain 9 billion by 2050, requiring more than 70% of the food production to satisfy that population growth demand. Therefore, efficient management of resources to improve agricultural production is of vital importance. In that vein, there is a need for knowledge on how phosphorus (P) regulates the diverse physiological and molecular mechanisms, and how it is fundamental for developing stress-tolerant and high-yielding varieties for efficient P resources management, particularly under the conditions of abiotic stressing factors. At the plant cellular level, P is a crucial element for various physiological and biochemical functions. It enters a wide range of metabolic processes, specifically, the synthesis of nucleic acids and energy generation of plants which makes it hard for plants to grow under P starvation (Carstensen et al., 2018; Malhotra et al., 2018; Powers et al., 2020). The deficiency of inorganic phosphate (Pi) in soil impairs fruit production and quality traits during the plant vegetative growth cycle (Li et al., 2021). Moreover, P plays role in vigorous root system formation and development, ultimately crop yields (Bén et al., 2015; Sun et al., 2016). However, in many farming contexts, many soils are P deficient and this deficiency severely limits crop yield and constitutes a global food security menace (Weikard, 2016; Heuer et al., 2017).

The application of soluble Pi in the form of phosphorus pentoxide (P_2_O_5_) during the plant cycle is highly recommended and widely applicable by growers during routine agricultural practices to supply the plant with the required Pi (Pang et al., 2018; Bindraban et al., 2020; Leitner et al., 2020). The supply of P as fertilizers is a quick and conventional method to address P deficiency. Such a method is regularly conducted in high-input agricultural systems, reaching application rates ~120 kg P ha^−1^ annually which depends on the crop species/varieties, productions, and agricultural management systems (Menezes-Blackburn et al., 2018). Through regular P supply in many intensively agricultural produced soils, there have been excessive P loads in soils over the last decades that may constitute a valuable pool of P legacy if appropriate cropping systems and management interventions are incorporated (Rodrigues et al., 2016; Lou et al., 2018; Teng et al., 2020). The crop production is often reduced by Pi deficiency under a low-input farm production system. Several biotic and abiotic factors limit the access of the plant roots to Pi (Jemo et al., 2015). The roots Pi uptake depends on the various physical, chemical, and biological factors of soil characteristics. This uptake is also negatively influenced by the abiotic stresses, specifically pH, organic matter, redox potential, the concentration of iron (Fe), Aluminum (Al), and calcium (Ca) (Sugihara et al., 2016; Young and Ross, 2018). In acidic soil, the soil Pi concentration is strongly pH-dependent, and Pi molecules interact with Fe and Al oxides and become low soluble for roots uptake (Penn and Camberato, 2019). Under alkaline soil conditions with a pH above 8, the Ca concentration is often dominant and rapidly reacts with Pi, which causes the formation of insoluble precipitates such as hydroxyapatite and a decrease in P availability (Yan et al., 2018; Penn and Camberato, 2019). Once applied to the soil, plants only absorb a small portion, whereas the highest proportion of Pi becomes less available due to adsorption or precipitation processes above-mentioned (Gérard, 2016; Lemming et al., 2019). Plant strategies to acquire Pi more efficiently or to mobilize soil-Pi from less accessible pools by the roots are required to increase P use efficiency under such circumstances (Jemo et al., 2006). Such strategies may involve improved Pi transport and soil/root contact through lateral root formation (Jia et al., 2018), increasing root-hair length (Zhang et al., 2018). Plant roots intimate beneficial interactions with soil microbes, such as growth-promoting bacteria or arbuscular mycorrhizal fungi (AMF), to further increase Pi uptake (Smith and Read, 2008; Saia et al., 2020). The incorporation of crop plant species with enhanced ability to release hydron (H^+^) or hydroxide (OH^−^), organic acid anions, and high production of rhizosphere phosphatase activity can benefit from increased poorly available inorganic and organic Pi fractions and contribute to plant P nutrition (Wu et al., 2018; Spohn et al., 2020; Wang and Lambers, 2020). Legume cover plants that are efficient at acquiring P from less available sources combined with cheaper Pi sources such as phosphate rock (PR) to increase P use efficiency accounts as part of improved agronomic practices (Jemo et al., 2006; Hallama et al., 2019). Review studies describing the role of cover crops in P recycling and their promising effects for sustainable intensification of agriculture management have been conducted (Hallama et al., 2019). The enhancement in soil-Pi has induced mechanisms, plant residues decomposition, improved physical properties, Al-detoxification from roots, and higher mycorrhizal root colonization development as the underlying benefits (Jemo et al., 2006; Ngome et al., 2013; Hallama et al., 2019).

With growing menaces of climate change, the adverse impacts of various stresses on crops are likely to aggravate global food security demand (Lamaoui et al., 2018; Bhuyan et al., 2019; Sarkar et al., 2019; Siddiqui et al., 2019). Soils altered by salts, drought, acids, or heavy metal stresses are potential reserves for arable lands to be re-used for agricultural purposes if well-managed. Development of innovative technologies to improve P use efficiency and management is needed. Technology involving the use of microbes to Pi solubilize, partially activated PR, development of slow/controlled Pi fertilizer, the nano-scale, and foliar fertilizer formulations could play an important role. The objective of the present work was to review the role of Pi molecules in the regulation of plant growth and abiotic stresses mitigation such as drought, salinity, and heavy metals. Specifically, we aimed at the following:

- Evaluating to what extent a P well supplementation or starvation condition can cause changes in the photosynthetic functions
- Examining the change in plant growth development under the conditions of well-P and low-P supply from peer-reviewed articles
- Investigating whether plants well supplemented with P grown in a combination of another abiotic stress, improve this stress tolerance and the underlined induced mechanisms.
- Examining the novel discovered genes underlining the Pi-dependent abiotic stress regulations
- Assessing agronomic available Pi-based interventions that could be rapidly integrated into farming practices use to mitigate Pi or different other abiotic stress in combination or alone.

## P as a Key Element for Plant Growth and Metabolisms

### Pi Is a Tricky Nutrient for Plants

Phosphorus is the second essential macronutrient required for plant growth and development alongside nitrogen (Roch et al., 2019). P is involved in various metabolic functions and many living cell regulatory processes are P-dependent (Razaq et al., 2017). The molecules of P constitute the structural skeleton of other biomolecules such as ATP, NADPH, nucleic acids, phospholipids, and sugar-phosphates for the primary and secondary plant metabolisms (Zhang et al., 2009; Stigter and Plaxton, 2015; Lambers and Plaxton, 2018). In many soils, the total soil-P content is often in the range of 400–1,200 mg kg^−1^ in the form of apatite and other primary minerals (Bindraban et al., 2020). Less than 0.1% of the total P exists in inorganically (Pi) available forms for plant uptake due to low solubility of Pi, slow diffusion, and high soil reactivity (Walpola, 2012; Menezes-Blackburn et al., 2016). It is worth mentioning that the Hedley fractionation procedure determines the amount of Pi and organic phosphorus (Po) in various soil extracts (Hedley et al., 1982; Cross and Schlesinger, 1995). The critical Pi values in soil that are readily available for root uptake to complete their life cycles are in the range of 10–15 mg P kg^−1^. These values are determined either by the Olsen or the Mehlich-III methods depending on the nature of soil (Olsen et al., 1954; Mehlich, 1984). Depending on ions concentration and soil pH, the soil Pi is often associated with Fe and Al for acid soils or with Ca in calcareous soil conditions, making Pi sparingly available to plant roots (Mishra et al., 2017).

The Po is produced from the metabolic activities of living cells, representing up to 80% of the total P (Mueller et al., 2019). Inositol phosphate, a dominant class of Po in the soil, is also known as phytate and synthesized by plants and is strongly complexed with soil compounds (Haygarth et al., 2018). Other Po compounds are orthophosphoric acid identified as inositol phosphates, phospholipids, and nucleic acids. In soils, the bioavailability of P is very low, reaching only 1 mg kg^−1^ of soil absorbed by plants as orthophosphate ions. Due to its higher mobility, available Pi to roots is often insufficient in arid neutral and acidic soils and limits plant growth (Schelfhout et al., 2021).

### Factors Regulating P Availability in Soils and Plant Uptake

#### Physical Factors

The dissolution of Pi compounds depends on various physical factors such as soil texture and moisture (Sugihara et al., 2016; Young and Ross, 2018). The particles size of soils influences the availability of mineral elements, in particular Pi (Sugihara et al., 2016). When the clay content increases, the Pi retention in the soil also increases and less Pi is available in the solution (Pizzeghello et al., 2016). Likewise, aeration or soil compaction could also affect the supply of roots of the necessary oxygen. In compacted soils, the oxygen circulation is limited and reduces the Pi diffusion near the root area (Silva et al., 2018). Water availability influences the Pi diffusion into the soil solution and the relative Pi for plants uptake (Young and Ross, 2018).

#### Chemical Factors

Various soil chemical factors interact with each other and affect the Pi availabilities and diffusion in soils, specifically pH, organic matter, redox potential, and the concentration of Fe, Al, and Ca (Fink et al., 2016; Moreira et al., 2017; Torri et al., 2017). The soil Pi concentration is strongly pH-dependent (Penn and Camberato, 2019). Under alkaline soil conditions with a pH above 8, the Ca concentration is often dominant in soils and rapidly reacts with Pi and causes the formation of insoluble precipitates such as hydroxyapatite that leads to a decrease in P availability (Yan et al., 2018; Penn and Camberato, 2019). On the contrary, soil acidity also has a negative impact on the availability of Pi. Soil acidity above ≤ 5.5 plays a negative impact on the Pi by interacting with exchangeable Al- and Fe- and forming Al-Pi and Fe-Pi complexes, that become less accessible for roots uptake (Penn and Camberato, 2019). Organic matter, especially hummus, can also interfere with P binding sites, making P available, rather than being bound to other metals (Fink et al., 2016). Soil organic matter is an important P source as its decomposition process increases P availability for plant uptakes (Horta et al., 2018).

#### Biotic Factors

Microorganisms are the central actors in the nutrients release and recycling process in plants (Jing et al., 2017). Besides, microbial roles in nutrient release, root activity account for a continuous release of free ions, oxygen, water, enzymes, and primary and secondary metabolites that directly affect the microbial diversity and influence the Po release in the rhizosphere (Wang and Lambers, 2020). The work of Badri et al. (2013) reported that the application of phytochemicals, mainly phenolic compounds from *Arabidopsis* roots were able to stimulate or inhibit different bacterial communities. While the study of Eisenhauer et al. (2017) observed that the diversity of root exudates improves soil fungi over bacteria microbiota. These modifications in the microbial structure around the rhizosphere might impact the P availability in the soil as the dominance of certain communities eliminates other species that could solubilize soil Pi. On the other hand, microorganisms promote the availability of Pi in the soil through the production of organic acids to release P complexed into Al, Fe, and Ca molecules (Kalayu, 2019). Various studies have identified the diversity of organic acids produced by microorganisms and their role in Pi solubilization activities (Kalayu, 2019; Wang and Lambers, 2020). Under *in vitro* conditions, organic acids decrease pH values, and acidification of microbial cells leads to the release of P by substitution of H^+^ by Ca^2+^ ions are postulated as an underline mechanism (Wang and Lambers, 2020). Besides, the production of more than one variety of organic acids has a synergetic effect on a greater Pi release in soil (Marciano Marra et al., 2012). Other inorganic acids such as chloric acid, nitric acid, and sulfuric acids produced by chemoautotrophs, as well as the role of H^+^ pump especially in *Penicillium rugulosum*, contribute to actively mobilize the complex Pi forms in soil (Richardson et al., 2011; Kalayu, 2019). The release of inorganic acids dissolves the tricalcium phosphate into di- and monobasic Pi and makes better P available for plants. Furthermore, the microbial assimilation of ammonium (${\text{NH}}_{4}^{+}$) is accompanied by the release of protons, considered as an effective mechanism for Pi solubilization in soil without requiring a demanding process such as organic acids production from the microbial cells (Alori et al., 2017).

Soil management practices such as legume cover inclusion in cropping system, conservation tillage practices are also efficient interventions to increase P use efficiency and influence the Pi availability (Jemo et al., 2006; Hallama et al., 2019). Additional benefits are reported by the PR fertilization of the preceding legumes crops, providing that the root exudation from legumes will solubilize the PR and increase the Pi (Jemo et al., 2006). The enhancement of soil physical properties, suppression of soil-borne diseases and enhanced mycorrhizal colonization promotion, and soil health benefits are the significant benefits observed in addition to increasing available Pi in soil (Hallama et al., 2019). Plant-associated plant microbiome and roots drive the Pi and Po solubilization under environmental conditions. Additionally, the Pi availability in soil shapes the microbial diversity. For instance, mycorrhizal colonization and species diversity are often inhibited under higher Pi concentrations, while low-Pi conditions stimulate their efficacy (Nouri et al., 2021). However, the critical Pi threshold that determines optimal microbiome diversity and functionalities for optimal plant growth is under-investigated (Nouri et al., 2021).

Climate change uncertainty is likely to accelerate the rate of P decomposition and P nutrition of plants owing to elevated carbon dioxide and temperature. Elevated carbon dioxide (CO_2_) and temperature could affect plant utilization and the acquisition of Pi and management to maintain ecosystem sustainability in P-deficient regions by increasing the rate of decomposition in soil, stimulate photosynthesis, and subsequent growth responses (Jin et al., 2015; Wang W. et al., 2020). Elevated CO_2_ and/or temperatures may alter P acquisition through changes in root morphology and increases in rooting depth (Qiu et al., 2021). Furthermore, the quantity and composition of root exudates are likely to change under elevated CO_2_, and/or temperatures may affect the carbon fluxes along the glycolytic pathway and the tricarboxylic acid cycle (Zayas-Santiago et al., 2020). The alteration of the plant biochemical environment due to microbial activities and root exudation changes will accelerate the Pi solubilization in the rhizosphere owing to elevated CO_2_ and temperature conditions (Jin et al., 2015). On the soil continuum, elevated CO_2_ and temperature will cause rapid organic matter decomposition and influence the soil Pi availability (Jia et al., 2020). However, most of the free-air carbon dioxide–enrichment and elevated temperature studies had only focused on rice and wheat plants (Ainsworth and Long, 2021).

## The Approach

In this study, we conducted a systematic analysis of retrieved published peer-reviewed articles to generate evidence from the Pi effects on physiological, growth, and mitigation of adverse stressing factors in plants. A literature search on the P, physiological functions, growth and development, and abiotic stress regulations was performed using Web of Science from Clarivate Analytics (webofknowledge.com; United States), Google scholar from google (https://scholar.google.com; United States), and Scopus from Elsevier (https://www.scopus.com/home.uri; Netherlands) platforms to curate articles published until 2021. Our keyword search included the terms “Pi application,” “photosynthesis,” “growth,” or “abiotic stress.” The screening of the searched articles by titles, authors, and abstracts helped to eliminate redundant articles from the database. Data were extracted using the following criteria. First, on P application, the study includes sufficient- and deficient-P supply. Second, on the photosynthesis traits, observations from studies on the total chlorophyll, rate of photosynthesis, stomatal conductance, and leaf area were reported under sufficient-P and deficient-P conditions for soybean (*Glycine max*), cowpea (*Vigna unguiculata*), wheat (*Triticum aestivum*), and maize (*Zea mays*) plants (Supplementary Table 1). Third, on plant growth traits, observations on the plant growth (shoot and roots), internal shoot, and root-P concentrations were used for the same species (Supplementary Table 1). Plant traits under the abiotic stress were categorized based on the stress nature and plant species. From drought or water, the shoot and P concentration and the pectin membrane expression modulated by P supplementation were considered for soybean, cowpea, wheat, and maize species. Fourth, on the heavy metal component, shoot-Cd, -Pb, and -zinc (Zn) accumulation in shoots from wheat, sedum (*Sedum alfredii*), rice (*Oryza sativa*), barley (*Hordeum vulgare*), maize, and rapeseed (*Brassica napus*) species (Supplementary Table 1). Due to the heterogeneity from the reported studies, the data were normalized by conversion from all reported units to international standard units. Therefore, the total chlorophyll content was reported as mg g^−1^ Fwt; the rate of photosynthesis as μmol CO_2_·m^2^·s^−1^, the stomatal conductance was reported as mmol·m^−2^·s^−1^, and the leaf area per cm^2^. The dry biomass of shoots and roots were reported as DM g plants^−1^. The shoot- and root-P concentrations were reported in percentage (%) of analyzed tissue-P. The decrease or increase due to the P effect was calculated using the following equation (Equation 1):


(1) $$\begin{array}{l} \text{P effect }=\;[(\text{P sufficient supplied value}-\\ \text{ P deficient supplied value})\text{/}\\ \text{ P deficient supplied value}]\times 100. \end{array}$$

The Shapiro-Wilk and Levene's tests were conducted to check for their normality and variance homogeneity from the data. Data with the satisfied normal distribution range (from −1.28 to 044 and *p* > 0.05) according to Kolmogorov-Smirnov analysis were used for the boxplots with values with 95% confidence interval. The means/PDIFF test was run to compare the pair-of treatment means. Means followed by the same letters in the figures are not significantly different at ^*^p < 0.05 according to Student–Newman–Keuls significant difference test.

## Pi-Dependent Regulation of Photosynthesis

**Figure 1:**
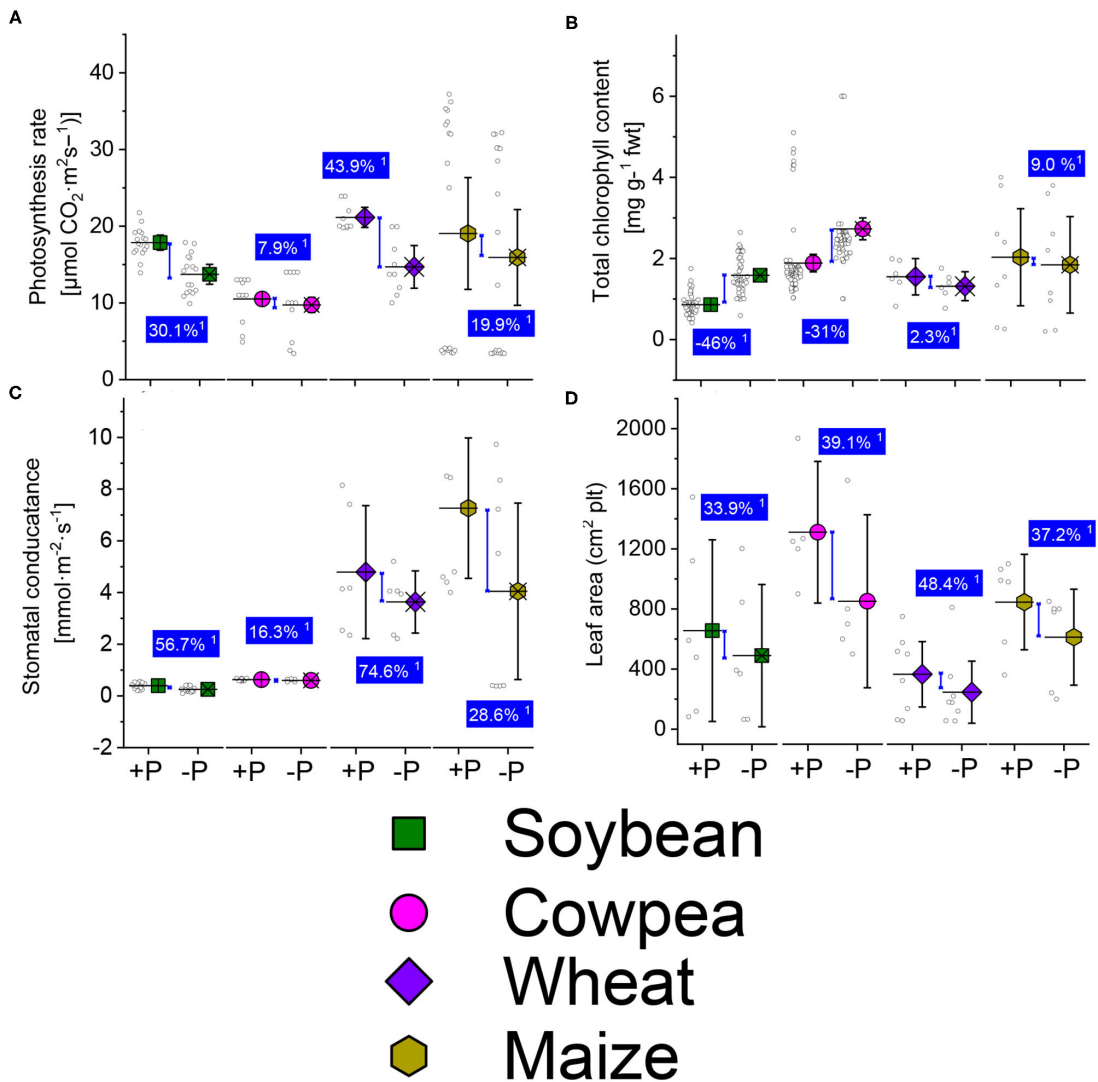
Boxplots and scatter data of the photosynthesis rate **(A)** total chlorophyll content **(B)** stomatal conductance **(C)** leaf area **(D)** under sufficient-P (+P), and deficient (–P) conditions of soybean, cowpea, wheat, and maize plants. ^1^ Average change (%) of +P applied over P-deficient plants. Means + bootstrap at 95% CIs of two tested P treatments that do not overlap indicate a significant relative increase difference.

The net photosynthesis rates of sufficient-P treated were higher than in the P-deficient ones under soybean, cowpea, wheat, and maize plants (Figure 1A). Average net photosynthesis rates increase due to P supply over the P-deficient treatment were 30.1, 7.9, 43.9, and 19.9% under soybean, cowpea, wheat, and maize plants (Figure 1A). The total chlorophyll contents under P deficient legume crop species were significantly higher in the P-deficient plant than the well-P treated, showing a reduction of −46 and −31% under soybean and cowpea plants, respectively (Figure 1B). The stomatal conductance change rate between the well-P supply and P -deficient plants were 56.7, 16.3, 74.6 and 28.6% under soybean, cowpea, wheat, and maize plants (Figure 1C). The leaf area (Figure 1D) was not statistically different between the well-P and the P-deficient plants.

**Figure 2:**
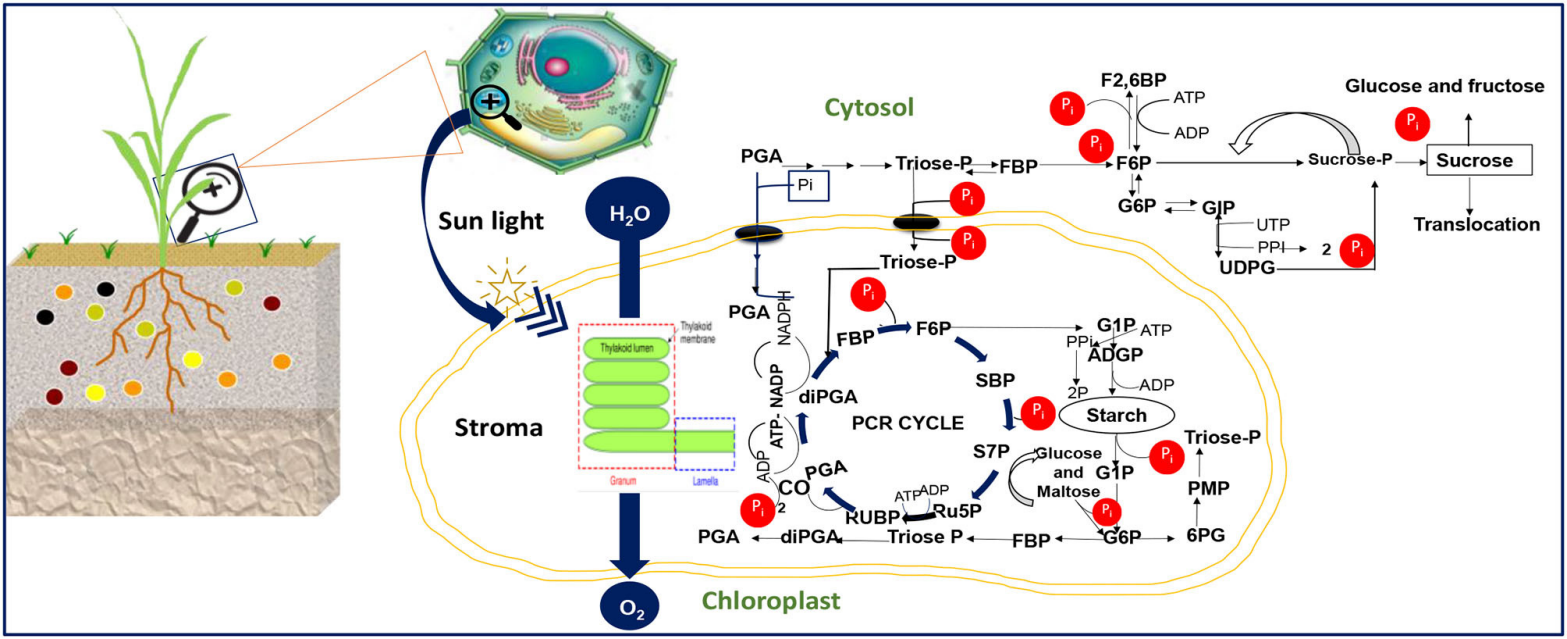
Cellular Pi-dependent regulation of the plant photosynthesis activity displaying the major process. PGA, 3-phosphoglycerate; diPGA, 1,3-diphosphoglycerate; FBP, Fructose-1,6-bisphosphate; F2,6, Fructose-2,6-bisphosphate; F6P, Fructose 6-phosphate; Ru5P, Ribulose-5-phosphate; RuBP, Ribulose-1,5-bisphosphate; SBP, Sedoheptulose-1,7-bisphosphate; S7P, Sedoheptulose-7-phosphate; G6P, Glucose 6-phosphate; G1P, Glucose 1-phosphate; Triose-P, Triose phosphate; ADPG, ADP-glucose UDPG, UDP-glucose; PMP, Pentose monophosphate; 6PG, 6- phosphogluconate; ATP, Adenosine triphosphate; ADP, Adenosine di-phosphate; NADP, Nicotinamide adenine dinucleotide phosphate; UTP, Uridine-5′-triphosphate; PP, pyrophosphate. The image illustration was redesigned by NB and MJ.

Our aggregated data evidenced a reduction in the net photosynthesis, stomatal conductance, and leave area trait under P starvation conditions (Figures 1A,C,D). The chlorophyll pigment accumulated in plant cells under P starvation conditions, in revanchist (Figure 1B). Figure 2 presents the mechanisms for photosynthesis and sugar formation in higher plants and the role of Pi in the regulation. The Pi interferes in several growth processes during the vegetative cycle. High energetic molecules, ATP, and NADPH are prerequisites to ensure photosynthesis functioning during the Calvin cycle (Hashida and Kawai-Yamada, 2019; Chen et al., 2020). The process can be divided into three important phases, namely carboxylation, reduction, and regeneration (Figure 2). During the carboxylation phase, CO_2_ and water molecules combine in the presence of Ribulose-1,5-bisphosphate carboxylase-oxygenase [Rubisco enzyme, with ribulose-1-5-biphosphate (RuBP)], and form 2 molecules of the 3-phosphoglycerate (3-PGA). Subsequently, the 3-PGA molecules are reduced to triose-phosphate (glyceraldehyde 3-phosphate) by NADPH and ATP molecules (Figure 2). At the latest step of the process, a major part of the trioses-P is used in *de novo* the RuBP biosynthesis using ATP, making it possible to restart the cycle again while the other parts are going to sugar synthesis (Figure 2). A positive or negative change of Pi concentration in the stroma affects the enzymatic activity of RuBisCO and fructose-1,6-bisphosphatase and the efficiency of the cycle. Phosphorylated derivatives such as RuBP, triose-P, PGA, G6P, and nucleotides decline in response to Pi starvation (Malhotra et al., 2018). In the case of Pi starvation in the process, the product from photosynthates immediately converts to starch biosynthesis, as a plausible explanation (Figure 2). The RuBisCO activation and the proper functioning of the Calvin cycle are then sensitive to Pi starvation. Furthermore, low Pi content in the chloroplast limits the ATP/ADP ratio by reducing photophosphorylation limits the cycle efficiency (Malhotra et al., 2018).

## Pi as a Key Element for Plant Growth and Metabolism Regulations

**Figure 3:**
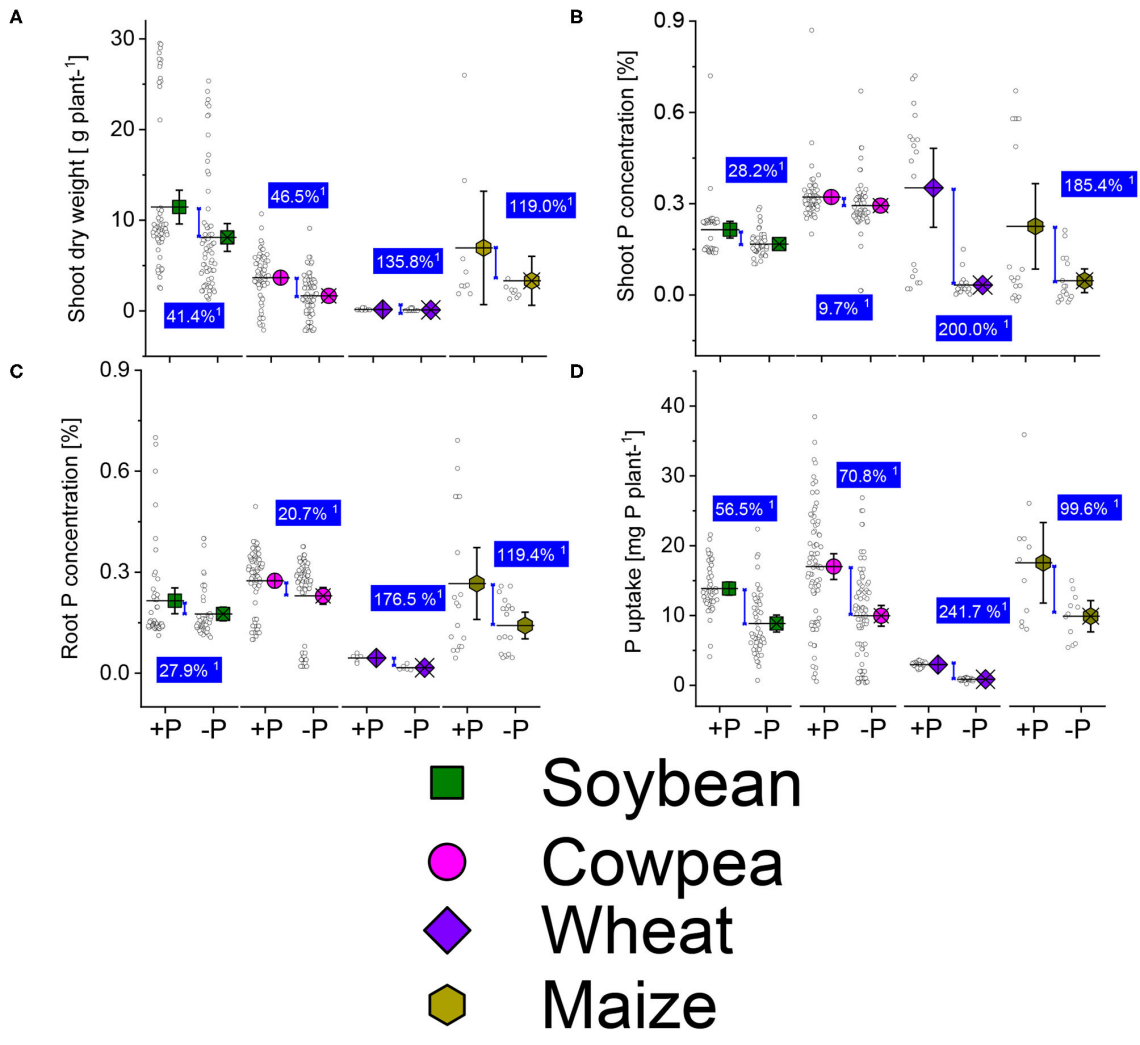
Boxplots and scatter data of shoot dry weight **(A)** shoot-P **(B)** root-P **(C)** concentrations, and P uptake **(D)** under +P and −P conditions of soybean, cowpea, wheat, and maize plants. ^1^Average change (%) of the sufficient-P applied over P-deficient plants. Means + bootstrap at 95 % CIs of two tested P treatments that do not overlap indicate a significant relative increase difference.

The average shoot dry weight (Figure 3A) was lower in P deficient plants than in the sufficient-P supplied of soybean, cowpea, wheat, and maize from the different published studies used. The dry matter biomass reduction was 41.4, 46.5, 135.8, and 119%, higher in the P deficient plants than in the well-P supplied ones of soybean, cowpea, wheat, and maize crops. The shoot-P concentrations (Figure 3B) also decreased under P starvations showing a moderate reduction (26.2%) in soybean, weak reduction (9.7%) under cowpea, and higher reduction contents (200 and 185.4%) under wheat and maize deficient-P plants. About the root-P concentrations and P uptake, there was a significant difference between the well-P supplied and P deficient cereal crop plants (Figures 3C,D). About legume plants, the decrease in root-P concentration between the well-P and non-P deficient were 27.9 and 20.7% under soybean and cowpea plants (Figure 3C). The average increases due to P supplementation in P uptake were 56.5 and 70.6% over the P starved plants (Figure 3D).

The cereal crops were the most affected by the P starvation than the legumes (Figures 3A–D). P deficiency in maize decreases biomass production due to a reduced leaf size (Plénet et al., 2000). In beans, Pi deficiency is characterized by short, thin shoots, dull leaves with early loss, and reduced flowering and nitrogen fixation capacity (El-Tarabily et al., 2008; Sanz-Saez et al., 2017). We should bear in mind that the data used in the present reviews came from experiments of different nature, various sources of P sources and rates, climatic conditions, experimental setup, and possible managements interventions, varieties/ genotypes from each crop (Supplementary Table 1). These could introduce bias in the interpretation of the results. To resolve the tissue-P imbalance, P is remobilized from old plant organs (roots, shoots, and leaves), translocated, and stored into seeds during filling stages (Plénet et al., 2000). The P contained in the seeds constitutes an important supply for the plants at the germination stage (Khan et al., 2018). Greater P concentration is observed in young leaves at earlier stages of plant growth for better leaves formation and role in cell division which stimulates the growth of leaves and roots in size and depth (Kavanová et al., 2006; Malhotra et al., 2018). On soybean (*G. max*), low-P supply reduces the number of flowers, while increasing P supply improved the number of pods and yields (Fernández et al., 2007).

**Figure 4:**
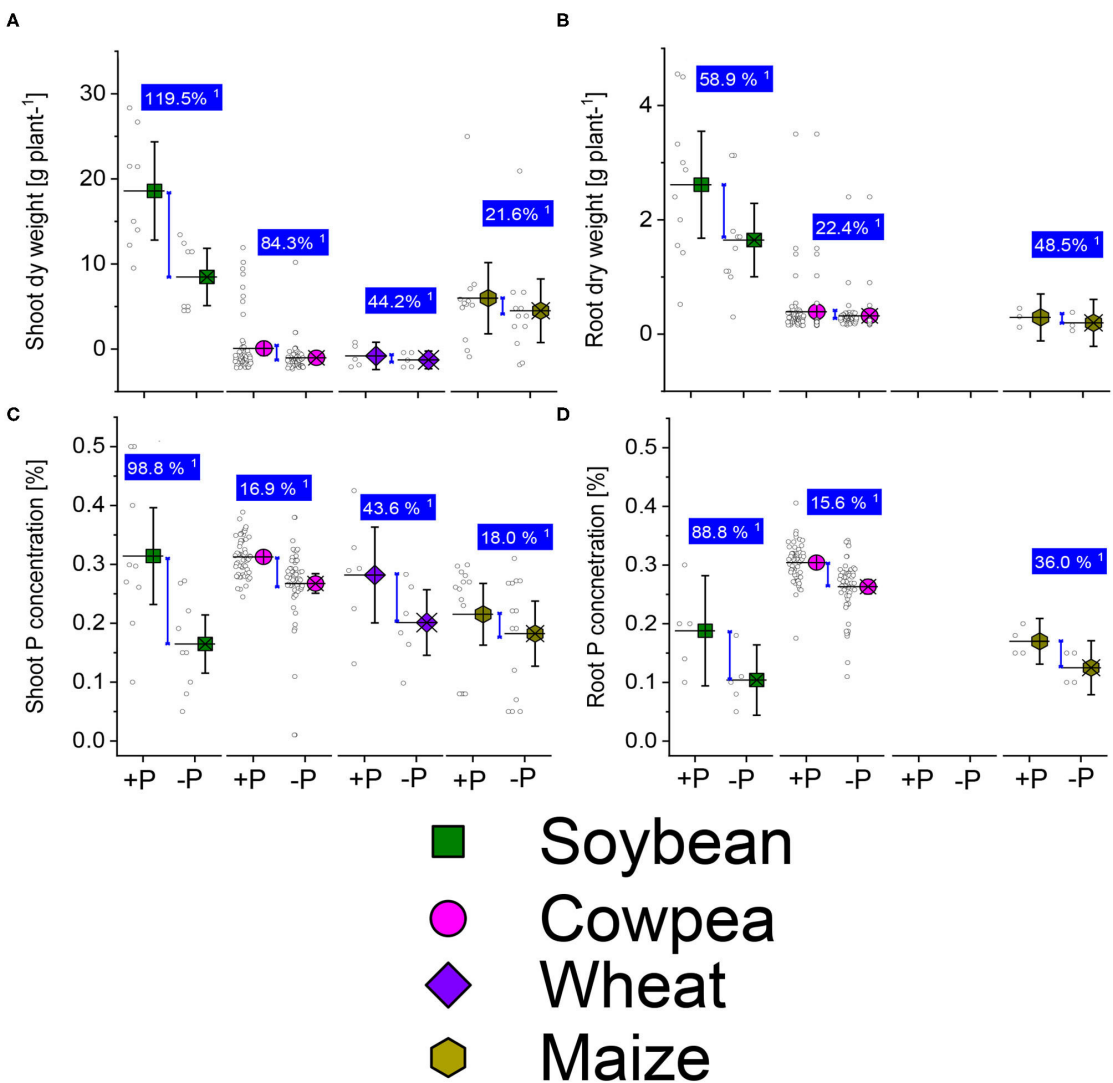
Boxplots and scatter data of shoot **(A)** root **(B)** dry weights, shoot-P **(C)** and root-P **(D)** internal concentrations under +P and −P under drought or water deficit conditions of soybean, cowpea, wheat, and maize plants. ^1^Average change (%) of the +P applied over P-deficient plants. Means + bootstrap at 95% CIs of two tested P treatments that do not overlap indicate a significant relative increase difference.

The mechanism underpinning Pi regulation engages a complex set of coordinated reactions from its absorption from the soil phase to the plant utilization (Figure 4). Pi is involved in many metabolic functions of plants and regulates many processes (Razaq et al., 2017). A decrease in the P concentration induces alternative pathways other than the conventional metabolic process, particularly the Pi-dependent glycolysis and mitochondrial respiration regulation at the cytoplasmic level (Le Roux et al., 2014). Pyruvate generation occurs from malate synthesis *via* malate dehydrogenase and mitochondrial malic enzyme (Figure 4). These alternative pathways that are P-dependently regulated, ensure the flow of carbon, electrons, mitochondrial respiration in conditions of P deficiency stress (Le Roux et al., 2014). P deficiency also affects cell division owing to change in cell divisions in the meristem, and the reduction of mitosis rates (Kavanová et al., 2006; Carstensen et al., 2018). On the opposite side, adequate P supplementation in plant roots promotes cell division and increases plant growth (Razaq et al., 2017; Malhotra et al., 2018). The roots of plants under Pi-starvation excrete various enzymes such as phytase and phosphatase to increase Pi availability (Vengavasi and Pandey, 2018). In wheat plants, the study of Nguyen and Stangoulis (2019) observed significant changes in the metabolite profile in leaves and roots under low P supply. These variations in the plant behavior under Pi starvation could generate the most tolerant cultivars that resist P limitation.

## Role of Pi in Managing Abiotic Stress in Plants

### Drought or Water-Induced Stress

**Figure 5:**
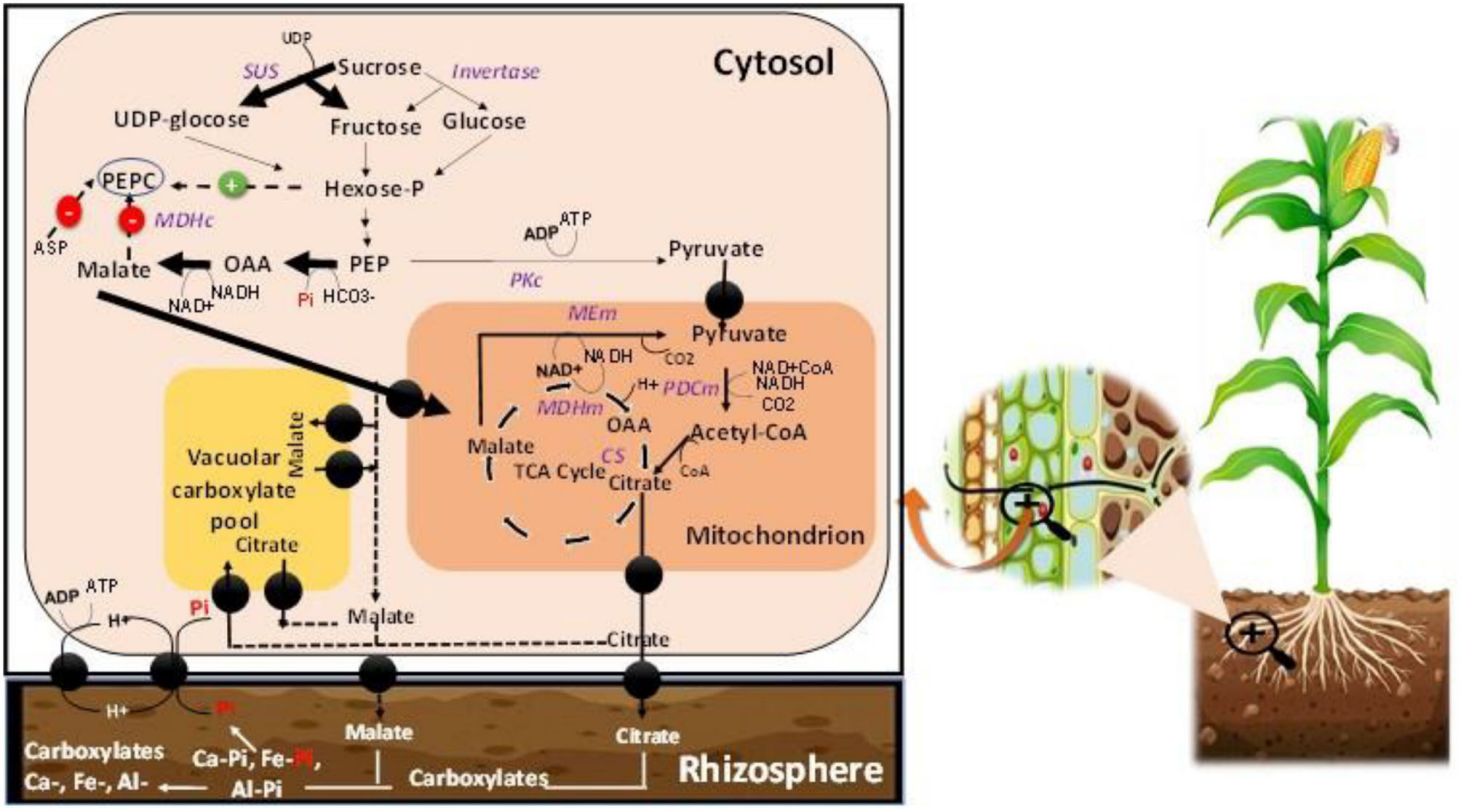
Pi-dependent regulation of the plant growth process at the cellular levels. UDP-glucose, Uridine diphosphate glucose; PEPC, Phosphoenolpyruvate carboxylase; Hexose-P, Hexose phosphate; OAA, Oxaloacetate; ASP, aspartate; PEP, Phosphoenolpyruvate; SUS, Sucrose synthase; PKc, Cytosolic pyruvate kinase; MDHc, Cytosolic malate dehydrogenase; MDHm, Mitochondrial malate dehydrogenase; MEm, Mitochondrial malic enzyme; PDCm, mitochondrial pyruvate dehydrogenase complex; CS, Citrate synthase. The image illustration was redesigned by NB and MJ.

We analyzed datasets for the P studies of sufficient and deficient-P treatments of imposed drought/water deficit stresses on soybean, cowpea, wheat, and maize plants from various world climatic regions, experimental conditions, different stages (intermittent, short term, or long-term). We analyzed the dry weight of shoots and roots, shoot-P and root-P concentrations change under well-P or low-P supplementation (Figures 5A–D). Also, the P sources varied from water-soluble P application, foliar P application, the P bounds molecules particles (Supplementary Table 1). We observed a systematic reduction of the shoot and root biomass, shoot-P, and root-P concentrations in P deficient plants of soybean, cowpea, wheat, and maize plants under drought stress conditions (Figures 5A–D). The Pi supplementation reduced the drought damages, and the responses were highly variable, depending on the plant traits and legumes species. Legume plants with well-P supplied exhibited the highest increase responses than the cereal plants about the shoot dry weight (Figure 5A). A higher shoot-P concentration average response was in the well-P supplied (96.8%) soybean than in the low-P applied treatments (Figure 5C). The cowpea plants displayed moderate increase response effects in the sufficient-P than in the low-P supplied plants under drought/ water stress about the shoot- and root-P concentrations (Figures 5C,D). Cereal plants observed a moderate average response to P application under water stress for the measured plant traits (Figures 5A–D).

The present results showed an overall reduction in biomass and internal P concentrations from the analyzed legume and cereal crops, without ignoring the complexity of aggregating datasets of independently conducted studies. A field study carried out by Hansel et al. (2017) aimed at evaluating the effect of triple superphosphate and tillage on soybean roots grown under drought conditions showed that the P-applied treatments significantly enhanced soybean root growth at deeper soil layers and improved its resistance to drought stress. Another study performed by Tariq et al. (2017) to investigate the effects of two levels of irrigation (well-watered and drought-stressed) and phosphorous (P) fertilization treatment on *P. zhennan* seedlings reported that P application had a positive effect on root biomass which improves its water-extracting capacity from the soil. The same authors outlined that the outcomes were not only due to improvement of the root system but also to decreases in malondialdehyde content as well as the up-regulation of chloroplast pigments, osmolytes, and nitrogenous compounds following a P supply. An improved P supply alleviated drought-induced growth photosynthetic damages in tobacco (*Nicotiana tabacum*) roots, by up-regulating antioxidant metabolism and osmolyte accumulation (Begum et al., 2020).

Drought damaging consequences to crops represents a severe constraint for agriculture, particularly in the arid and semi-arid regions of the world. Water stress alters the antioxidative defenses of plant because of cell dehydration and the accumulation of reactive oxygen species (ROS) compounds (Vurukonda et al., 2016; Duc et al., 2018). The production of ROS interferes with plant photosynthesis machinery and subsequently impair photosynthetic activity and decreased chlorophyll concentration in leaves (Becklin et al., 2016; Li et al., 2019). Drought and Pi deficiency stress often interfere with plant metabolism *via* various stress signals and hormonal changes that play essential roles in regulation processes under natural conditions (Jemo et al., 2017). The Pi supply to plants alleviates the drought stress owing to the downregulation of the synthesis of phytohormones abscisic acid (ABA) and indoleacetic acid (IAA), as well as ROS concentrations reduction in plant tissue (Begum et al., 2020). However, the role of P molecules in the drought stress-regulated mechanism is under-investigated. Improvement in crop productivity remains a real challenge to meet the food security demands of an ever-increasing population. Therefore, it requires a better understanding of how P molecules are involved in plant mechanisms to mitigate drought/water stress. Phosphate fertilization improves solute concentration in drought-stressed and well-P compared with no-P treated plants (Tariq et al., 2017). The accumulation of solutes in plants protects cells from drought by substituting the hydroxyl group for water which maintains membrane proteins (Hoekstra et al., 2001). Phosphorus supply could enhance the plant resistance capacity to water stress by enhancement of roots system and improve accessibility of a plant to a large spectrum of water and nutrients sources (Razaq et al., 2017).

### Heavy Metal Stresses

**Figure 6:**
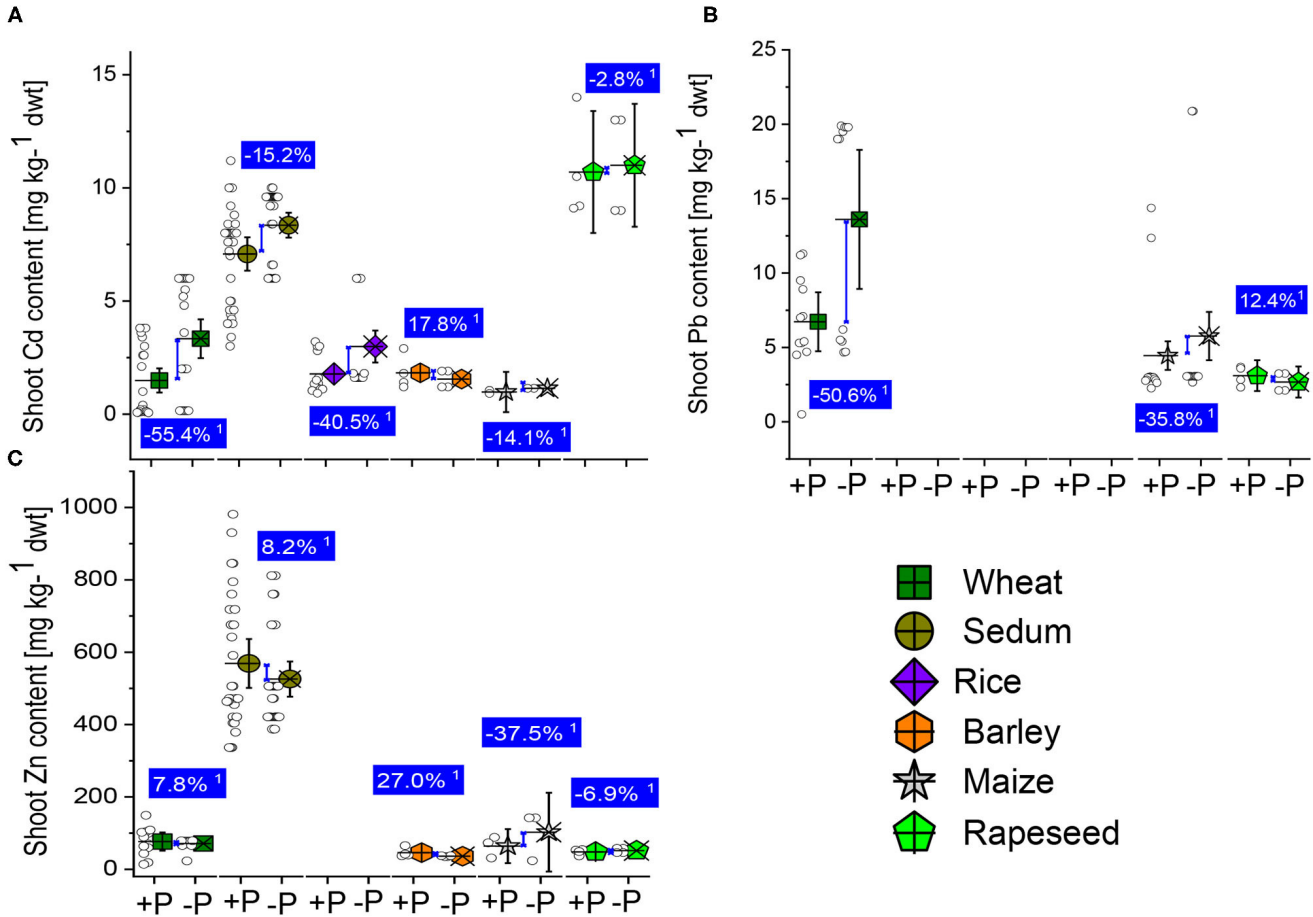
Boxplots and scatter data of shoot Pb **(A)** Cd **(B)** and Zn **(C)** contents under +P and –P conditions of wheat, sedum, rice, barley, maize, and rapeseed plants. ^1^Average change (%) of the +P applied over P-deficient plants. Means + bootstrap at 95% CIs of two tested P treatments that do not overlap zero indicate a significant relative increase difference.

Plants grown in soils contaminated with heavy metals experience a decrease or complete inhibition of their roots and shoot growth and development. The efficient P supplementation to the plants reduced the heavy metal concentration in leaves but cannot prevent plants from uptake the metals. Heavy metals compete with co-existing ions in soil and result in nutrient deficiencies (Zhao et al., 2018). We compared the concentration of Cs, Pb, and Zn in shoots between the well-P and low-P supply plants of wheat, sedum, rice, maize, and rapeseed (Supplementary Table 1). The shoot Cd and Pb accumulation significantly reduced by 55.6 and 55.4% in the sufficient-P than the low-P treated wheat plants, while the Zn content was not different (Figures 6A–C). We also noticed a reduction in Cd concentration in the shoot of sedum plants due to the P supplementation, while the Zn content slightly increased (Figures 6A,C). The shoot-Cd, -Pb, and -Zin concentrations were reduced in the well-P treated than in the low-P treated maize plants (Figures 6A–C). The decrease by 14.1, 35.6, and 37.5%, respectively, for the shoot Cd, Pb, and Zn concentrations. Due to the lack of sample size on the rice shoot Pb and Zn concentrations, we observed that the shoot Cd concentration reduced by 40.5% in the sufficiently-P treated plant than in the P-deficient maize plants. The P application effects on the shoot Cd and Zn concentrations were 17.8 and 27% (Figures 6A,C). On rapeseed, reduction by 2.8% and 6.9 in shoot Cd and Zn concentrations, while moderate increase by 12.4% in the shoot Pb reported in the well-P than in the low-P treated plants (Figures 6A–C). In a pot experiment carried out by Cao et al. (2008) to investigate the effects of different phosphate fertilizers on Pb uptake, the authors observed that Pi amendments reduced Pb concentrations in shoots and roots by 18.3–51.6% and 16.8–57.3%, respectively compared with the control samples. The superoxide dismutase (SOD) activity in the leaves further decreased significantly compared with the control. Similar observations were reported by Li et al. (2012) when investigating the effect of Pi enrichment of contaminated soil with Pb on the growth of ryegrass (*Lolium perenne* L. cv. *Aubisque*). In soybean, Cd affects the diversity, plant growth, and activity of rhizobia leading to a decrease in the number of nodules (Stan et al., 2011). High accumulation of Cd concentration in nodules cells accelerated senescence of nodules, reduces their number, and causes oxidative stress (Balestrasse et al., 2004; Sheirdil, 2012). Pi supplementation of crops often reduced the availability of heavy metals in soil from previous studies (Zeng et al., 2017; Cao, 2018). Finally, Gomes et al. (2013) submitted *A. Peregrina* plants to combined doses of arsenate and Pi. The addition of Pi considerably improved the phytoremediation capacity of *A. Peregrina* by increasing the accumulation of As. The Pi supply enhanced the catalase and ascorbate peroxidase activity and prevented lipid peroxidation in the leaves of A. *Peregrina* plants. Adequate P application could then constitute a way to immobilize metals in soils. However, the doses of Pi to supply to the plants are necessary to consider, and a reasonable and well-studied P to metal ratio would effectively immobilize metal toxicity from contaminated soil.

The supplementation of Pi in heavy metal contaminated soils form a complex compound that immobilizes the heavy metals and their unavailability and uptake by the root system (Zeng et al., 2017). Further, the use of Pi enhances plant growth parameters and decreases Cd and H_2_O_2_ concentrations in shoots. The stimulation of wheat antioxidant enzyme activities, which is often inhibited under Cd soils, is enhanced due to the Pi supplementation and preserves the cell membrane from damage caused by Cd (Arshad et al., 2016). Other mechanisms engage the synthesis of phytochelatin compounds that complex and compartmentalize Cd in plant leaves to reduce elevated Cd concentration in the cells. The process is highly coordinated by precursor molecules, Glutathione and P-dependent regulated during the biosynthesis process (Boukhalfa-Deraoui et al., 2019). The Pi enrichment of contaminated soil will then help the plants detoxify from heavy metal accumulation.

### Salt Stress

Soil salinization in irrigated lands is an increasing menace affecting global agricultural production and sustainable utilization of land resources, especially in arid and semiarid regions (Zewdu et al., 2017; Asfaw et al., 2018; Siddiqui et al., 2019). Effective management of salt stress to minimize environmental impact is imperative for the sustainability of irrigated agriculture. Salt stress interferes with root development and inhibits the growth at high sodium chloride (NaCl) concentrations. The reduction in nutrient uptake at early at the early growth stage of the plant is present (Hussain et al., 2017). The seed germination rate decreases from a concentration of 50 to 300 mM of alkaline salts (Na_2_CO_3_, NaHCO_3_), while a low concentration (50 mM) of neutral salt (NaCl, Na_2_SO_4_) and alkaline salt stimulated seed germination, showing a higher germination rate and longer radicles and hypocotyls than the control (Hu et al., 2018). Salt stress also delays the seed germination period to their complete inhibition due to insufficient water absorption by seeds or to ion toxicity (Siddiqui et al., 2019). Higher salt concentrations in seeds reduce the hydrolytic enzyme activity, such as amylases, proteases, and phosphatase (Nasri et al., 2016; Borzoui et al., 2017). Besides seed germination, the depressive effect of salts often depends on the used crop variety and/or cultivar, and the concentration of 25 mM of NaCl strongly depressed bean (*Phaseolus vulgaris)* plants (Faghire et al., 2012).

Most studies investigating the effects of salinity or P deficiency as a separate growth-limiting factor on plant growth and nutrient uptake, but the literature on their combined effects is still limited (Tang et al., 2019). The role of P in energy transfer and enzyme building and their regulation could be possible strategies for plants to cope with salt stress. Besides, salt excretion by the halophytes glands is an expensive process that requires ATPase enzymes and P-dependency (Assaha et al., 2017). Therefore, it is necessary to increase the availability of P to plants under salinity conditions for osmotic adjustment and growth (Miranda et al., 2012). The increase in Pi fertilization could balance the plant mineral uptake by increasing the nutrient availability lost through antagonism. It should be noted that increased sodium content decreases soil P availability. Miranda et al. (2012) reported that the P-amended plants had higher growth parameters than plants subject to saline stress without P fertilization under greenhouse conditions. The P supplementation attenuated the salt stress effects and improved soybean growth but did not eliminate the Na+ accumulation in root tissues (Miranda et al., 2012). However, in another study by Tang et al. (2019), they further pointed that a combination of salinity and high P does not exclude maize plants from the uptake of salt from solution, showing a two-fold higher Na+ concentration in the sufficient-P treated maize than the P deficient. The authors also observed that salt stress significantly increased shoot P concentration of maize with sufficient P (Tang et al., 2019).

The salt tolerance avoidance/acceptance mechanisms of plants under sufficient-P and P-deficiency conditions are under-investigated. The few studies on plant responses to salt stress and P supplementation often result in complex and divergent data interpretations. For instance, salt excretion from leaves is the most adaptive mechanism induced by salt stress alone (Flowers and Colmer, 2008). Under combined salt and high-P supply, the increase in shoot Na+ concentration was due to the changes in root physiology (Tang et al., 2019). In the leaf and root cells of *Zostera marina* plants, sodium-dependent high-affinity P transporter genes expression genes at the plasma membrane increased, indicating a synergistic effect may exist between Na+ and P uptake (Tang et al., 2019). To maintain a relatively low Na+ concentration and high K+/Na+ ratio under saline conditions and sufficient Pi supply, the extrusion of Na+ is also an important mechanism.

### Heat Stress

Heat stress occurs when the temperature increased 10–15°C above the ambient and affects crop growth and yield (Sarkar et al., 2019). The very quick short-term benefit from heat stress can be the increased plant metabolic activities. However, heat stress inhibits many pant functions, especially photosynthesis and results in plant lethality in tropical and subtropical regions (Sarkar et al., 2019). The study of Fahad et al. (2017) showed that the damaging effect of high temperature is due to the inability of plants to absorb nutrients and water and use them. This leads to a reduction in the number of plants sown per cultivated plot. In wheat, the damages from heat stress are prejudicial (Lamaoui et al., 2018). A better P nutrition could alleviate or reduce the severity of heat stress effects on plants. Furthermore, the work of Fahad et al. (2017) examined the influence of high-temperature stress combined with different biochar and P fertilization treatments on the growth, grain yield, and quality of two rice cultivars. The authors observed higher grain production under plants treated with biochar + P supply under heat stress due to enhanced photosynthesis, water use efficiency, and grain size of cultivated plants. Hence, Pi supply mitigated the adverse of high-temperature damage.

### Acid Stress

Soil acidity is adverse abiotic stress that had been under-considered for many decades. Soil acidity due to proton (H^+^) rhizotoxicity inhibits plant growth and development and globally limits crops (Bhuyan et al., 2019). In response to acid stress, plants increase the biosynthesis of organic acids such as oxalate or citrate capable of chelating Al and Fe attached to P molecules and their release for uptake (Yang et al., 2019). The pH modification around the rhizosphere to optimize P uptake is another mechanism in the plant-Al tolerance (Chen and Liao, 2016). Endophytic inhabiting microbes in P deficient soil also improve the P nutrition, exude phenolic compounds to complex exchangeable Al forms, and improve plant growth.

The dry biomass of shoots and roots increased in the Pi supplemented than the no-P treated wheat plants grown under acid soils conditions in an experiment conducted by Bhuyan et al. (2019). The P supplementation compensated harmful effects from acidity imply that Pi helped the plants to withstand acid stress (Bhuyan et al., 2019). Various P-induced mechanisms including the efflux of root Pi and exudation of ${\text{HCO}}_{3}^{-}$ or organic anions that complex with Al and improve Pi in the resulted contributed to increasing P uptake of wheat plants. In a study conducted by Opala et al. (2010) to evaluate the impact of organic and inorganic P sources on maize yields in acid soil conditions of Western Kenya, the authors observed that applied inorganic P increased corn yields and significantly reduced Al rhizototoxicity.

## Genes Involved in Pi-Dependent Regulation of Plant Stresses

Various Pi-starvation inducible genes in the Pi signaling pathway trigger different molecular responses to improve plant survival under Pi starvation conditions. In response to P deficiency, plants have developed several internal mechanisms to cope with Pi deficiency. These adaptive mechanisms induced by Pi deficiency are called the Pi starvation response. A proper description of candidate genes that mediate only the Pi uptake from soils into the plant and their translocation into chloroplasts, mitochondria, and Golgi apparatus have been extensively reviewed (Heuer et al., 2017). Table 1 depicts newly discover genes in the Pi alone or combined stress with Pi-starvation (drought and salinity).

**Table 1:** Names of genes, family, alleviated abiotic stresses, functions, isolated plant species, and cite references expressed in response to phosphate starvation, drought, and/or salinity stresses.

**Gene**	**Family**	**Alleviated Abiotic stress**	**Functions**	**Plant species**	**References in**
*LcPIP1;1 LcPIP2;1, LcPIP2;4; HvPIP2;2* and *HvPIP2;5*	*Plasma membrane intrinsic proteins*	Pi starvation and drought stresses	- Code for water channels (aquaporins) that facilitate the transit of water and/or other small solutes through cell membranes. - PIP genes are overexpressed in phosphate deficiency and/or drought which leads to abiotic stress alleviation.	*Hordeum vulgare* *Leymus chinensis*	Li et al. (2020)
*HvPIP1;3, HvPIP2;1*; *HvPIP2;2, HvPIP2;4*, and *HvPIP2;5*	*Plasma membrane intrinsic proteins*	Pi starvation and drought stresses	Code for water channels (aquaporins) that facilitate the transit of water and/or other small solutes through cell membranes. PIP genes are overexpressed in phosphate deficiency and/or drought which leads to abiotic stress alleviation.	*Hordeum vulgare* *Leymus chinensis*	Armand et al. (2019)
*PHO1; H1/4, PHO1*; *H5, PHO1; H8*, and *PHO1; H12/14*	PHOSPHATE1 (*PHO1*)	Pi starvation and salt stresses	- adaptation and morphological divergence - Salt stress tolerance	*Glycine max*	Wang et al. (2019)
*GmSPX-RING1*	*SPX-RING*	Pi starvation	-Control the Pi efficiency in different soybean cultivars	*Glycine max*	Du et al. (2020)
*GmPAP12*	*GmPAP*	Pi starvation	-Induce the synthesis of purple acid phosphatases as a strategy for nodules to acquire more Pi	*Glycine max*	Wang W. et al. (2020)
*GmPHR25*	*GmPT* Pi transporter	Pi starvation	-Up-regulated in response to Pi starvation is overexpressed in soybean hairy roots, with observed high-affinity to Pi transporter	*Glycine max*	Xue et al. (2017)
*GmETO1*	*Ethylene-Overproduction Protein*	Pi starvation	-Gene expressed notably for ethylene biosynthesis, and root development under phosphate deprivation	*Glycine max*	Zhang et al. (2020)
*PHT*	*PHT1*	Pi starvation	-Transport Pi from the soil to the different parts of the plant. -Ensures the transport of other P analog compounds such as nitrate, chloride, and sulfate		Roch et al. (2019)
PvSPX1–PvSPX3	*SPX*	Pi starvation	increased in the roots and leaves	*Phaseolus vulgaris*	Zhang et al. (2016)
*PHR1, microRNA399*, and *PHR1*	*PHR1*	Pi starvation	-Regulation of P homeostasis	*Arabidopsis thaliana*	Sun et al. (2016)

A recent study has highlighted new candidate genes expressed during exposure of plants to combined Pi starvation and drought stresses (Armand et al., 2019; Li et al., 2020). The regulation of aquaporins membrane (localized at the plasma membrane) that control water movement within/out of the plants and influence the hydraulic property of roots are affected. The expression of aquaporin genes coding for protein membranes responsible for apoplastic barriers synthesis decreased (Li et al., 2020). Specifically, the genes encoding the 3-Pi dehydrogenase bound synthesis *LcPIP1;1, LcPIP2;1, LcPIP2;4, HvPIP2;2*, and *HvPIP2;5* decreased. The reduced aquaporin genes expression significantly correlated with a decrease in root water conductivity. In turn, the root-to-shoot surface area ratio increased for additional resources remobilization to the roots to cope with drought stress (Li et al., 2020). However, in another study, the *HvPIP1;3, HvPIP2;1, HvPIP2;2, HvPIP2;4*, and *HvPIP2;5* genes were expressed by plants under both Pi and drought stress. The formation of apoplastic barriers increased considerably along the principal axis and lateral roots in low-nutrient treatments (Armand et al., 2019). These observations suggest that other family genes may account to control water movement during plants exposure to Pi and drought simultaneously could deserve further research investigations.

The *PHOSPHATE 1* (*PHO1*) gene family is among the Pi-starvation inducible genes involved in the Pi signaling pathway and trigger different molecular processes to improve plant survival under Pi stress in *Arabidopsis* plants (Wang et al., 2019). The gene was recently reported to play an important role in stomatal responses to ABA and a possible interaction among different signal transduction pathways in plants such as drought and salinity stresses (Wang et al., 2019). Under soybean plants exposed to high salinity stress, most of the *PHO1* genes were expressed and enhanced the stress tolerance at the high salt concentration (200 mM NaCl). Under Pi starvation, the *PHO1* genes (*PHO1; H1/4, PHO1*; *H5, PHO1;H8*, and *PHO1;H12/14*) were similarly upregulated in the roots of soybean genotypes between ZYD6 and SN14. This observation suggests that the *PHO1* gene family may play roles in soybean adaptation and morphological divergence to salt or Pi stresses (Wang et al., 2019). However, mechanisms of the soybean *PHO1* genes in relation to other various stresses, their crosstalk, and roles of adaptive evolution were still unclear, and the Pi-dependent regulation mechanisms need further investigations.

Other important studies were instigated many diverse genes involved only in the Pi signaling pathway that trigger P use efficiency (Du et al., 2020), nodule development (Wang W. et al., 2020), Pi homeostasis (Xue et al., 2017), and root growth development (Zhang et al., 2020) under Pi deficiency. The candidate gene *GmSPX-RING1* was over-expressed under soybean P deficient plants by negatively regulating soybean phosphorus concentration in hairy roots (Du et al., 2020). The *GmPAP12* gene is required to induce the synthesis of purple acid phosphatases as a strategy for nodules to acquire more Pi (Wang W. et al., 2020). The *GmPAP12* gene was highly expressed in soybean nodules that correlated with increased acid phosphatase and phytase activities under low P conditions (Wang W. et al., 2020; Wang Y. et al., 2020). The *GmPHR25* gene, up-regulated in response to Pi starvation is overexpressed in soybean hairy roots, with observed high-affinity to Pi transporter (*GmPT*) gene members (Xue et al., 2017). The ethylene-overproduction protein 1 *(GmETO1*) gene that regulates ethylene-biosynthesis and underlies the major QTL *q14-2* controlling Pi uptake was strongly induced under P deficiency, and overexpressed and significantly enhanced Pi deficiency tolerance by increasing proliferation and elongation of hairy roots, Pi uptake, and use efficiency (Zhang et al., 2020). The Pi absorption and its use by the plant are ensured by transporters (*PHT*) linked to the cell membrane and grouped into five families (Roch et al., 2019). *PHT1* family is designed to transport Pi from the soil to the different parts of the plant. It ensures the transport of other P analog compounds such as nitrate, chloride, and sulfate (Roch et al., 2019). The genes encoding these *PHT1* are Pi-stress sensitive and induced under Pi starvation. The legume and cereal genomes contain many members of the *SPX* gene family (Zhang et al., 2016). For instance, three SPX proteins (*PvSPX1–PvSPX3*) are present in common beans (*Phaseolus vulgaris*), and their expression levels increased in the roots and leaves under P starvation conditions (Zhang et al., 2016). The overexpression of the *PvSPX1* gene induced by P deficiency increases root P concentration and a change in the forms of the root hairs and lateral roots formation (Zhang et al., 2016). Furthermore, among other genes and proteins identified, several of these genes interfere in regulating P homeostasis, including *PHR1, microRNA399*, and *PHR1* that plays a central role in P signaling networks (Sun et al., 2016). The study of Devaiah et al. (2009) realized transcriptional analysis to investigate the biological regulation of *MYB62* induced under Pi deficiency. They found that the overexpression of *MYB62* resulted in altered root architecture, Pi uptake, and acid phosphatase activity. The collection of genes coding for signalization and Pi transporters and the crosstalk with other genes and pathways improve tolerance toward Pi starvation. All taken together, the *de novo* elucidated molecular mechanisms in the Pi signaling network, drought and salt tolerances, Pi-homeostasis are promising avenues to decipher the complex regulatory mechanisms of crop stresses tolerance. The enhancement of crop production in a pressing climate change context remains a global challenge to tackle.

## Agronomy Management Technological Strategies of P to Improve Plant Stress

Phosphorus-based agronomic technological interventions to increase P use efficiency and make Pi available for plants uptake under abiotic stresses under farmer field conditions exist or under development. A list of possible interventions including microbial-based, P-based fertilizer production, modes of application (soil or foliar), and the size dependent scale of nanoformulations. Table 2 summarizes the advantages and disadvantages of each technology with a focus on its scalability and affordability.

**Table 2:** Options/technologies, end-products, mode of actions, pros and cons of available P-based innervations to increase growth to mitigate P deficiency, enhancing P use efficiency in plants under abiotic stress conditions.

**Options/technologies**	**End-products**	**Mode of actions**	**Pros**	**Cons**
Microbial	Plant growth promoting rhizobacteria (PGPR) inoculants	- Access Pi - Mitigate the impact of drought - Promote growth - Fight against diseases	- Affordable	- Amount of Pi remain very low, - Difficult to scale up
	Mycorrhizal fungi inoculants	- Promote Pi availability and uptake - Mitigate abiotic stresses - Increase root volumes *via* mycelia - Induce the synthesis of low molecules such as trehalose	- Affordable - Amount of Pi could range 5–15 kg P ha^−1^	- Amount of Pi remain very low, - Difficult to scale up
Phosphate fertilizer	TSP, SSP granules or liquids forms	- Available Pi - Rapid access by plant roots - Plant access by root uptake - Soil application - Not always synchronized to the demands of plant at optimum request.	- Well-mainstreamed processes - Highly scalable - Popular at the marketplace	- Relatively low agronomy efficiency crops (20%) - Large application quantities - Fixation into soil particles - low mobility in the soil
	Activated RP granules	- Available Pi - slow/controlled releases - soil application - Favor microbial activitiesadd	- Amount of Pi remain very low, - Addition of other important nutrients - Easy to scale up	- Pi less soluble - Lack of sufficient research proven evidence
Pi fertilizer formulation and application method	Liquid Pi formulation	- Foliar spray - Plant uptake by - Stomata, epidermal structures as trichomes and lenticels uptake mechanisms	- Bypass root uptake - Increased efficiency of fertilizer use - Small quantity of Pi used - Economically affordable - Premium price	- Amount of Pi remain very low, - Difficult to scale up - Yield often unpredictable - Much research needed to value the approach
	Pi-nano scale particle size development	-Target delivery - Foliar spray - Plant uptake by - Stomata, epidermal structures as trichomes and lenticels uptake mechanisms	- small quantity of Pi used High mobility High energy transfer - Premium price	- Amount of Pi remain very low, - Difficult to scale up
	Foliar + Pi-nano scale particle size	- Pi available increase - Target Pi delivery Foliar spray - Plant uptake by - Stomata, epidermal structures as trichomes and lenticels uptake mechanisms	- small quantity of Pi used - Two complementary approach combined - Increase yield - Premium price	- Amount of Pi remain very low, - Not popularly used - Consumer behavior concerns

### Microbial Technology to Increase Pi

Under real field conditions, the management of Pi coupled with the use of beneficial microorganisms that inhabit the rhizosphere of many croplands can help plants to access Pi and to promote their growth and fight against diseases caused by pathogenic fungi, bacteria, viruses, and nematodes (Vejan et al., 2016). For example, plant growth-promoting rhizobacteria (PGPR) can help to mitigate the impact of drought on plants through the production of cytokinins which increase the synthesis of ABA in the leaves (Kaushal and Wani, 2016; Dubois et al., 2018). Generally, ethylene is formed from a precursor of 1-aminocyclopropane-1 carboxylate (ACC) and some PGPR bacteria such as *Achromobacter piecehaudii* produce ACC deaminases, which degrade ACC and reduce the accumulation of ethylene in plant tissue that induces systemic resistance against drought and salt and promotes plant growth (Glick et al., 2007; Etesami et al., 2020). Mycorrhizal fungi represent another important group of beneficial soil microbes to promote Pi availability and uptake by plants under abiotic stresses using several induced mechanisms. Apart from their proven role of increasing root volume *via* mycelia out the rhizosphere zone to access Pi a few centimeters distance away (Mathimaran et al., 2007), mycorrhizal plants are capable to induce the synthesis of low molecules such as trehalose stored as C in the mycelia and spores to improve the stress tolerance of the plant (Sharma et al., 2020). Importantly, trehalose levels increase under stress, as an energy source and are highly Pi- dependent regulated (Yao et al., 2018). However, for both PGPR and mycorrhizal approaches, most of the studies are often conducted under controlled conditions and fail to deliver the same promising results under real field contexts due to various challenges. Furthermore, the industrial scalability of the approaches often limits the potential of these technologies.

### Increase Pi From PR Acidulation

The sulfuric acid acidulation of PR is the quick and commercial method to make water-soluble Pi for soil application and subsequent plant uptake. The process offers industrial well-mainstreamed processes from raw material production, pretreatment, acidulation, and end-user products obtention. At the farm application stage, the relative agronomy efficiency by the crops does not exceed 20% of the amount applied and requires large quantities (Bindraban et al., 2020). During the entire vegetative period, the plants for Pi demand for soluble Pi forms may not always be in synchrony with its availability in the soil. Diverse biotic and abiotic factors reduce the Pi availability to the roots, such as the high soil fixing capacity, and the inadequate management by the smallholders at the on-farm level. Partial activation of PR, with possible highly acidic molecules that react with PR to breakdown the CaCO_3_ site, and gradually release Pi can contribute to tackling these problems by releasing a fraction of soluble Pi forms at the sowing and other fractions subsequently for slow/controlled release forms (Mao et al., 2017). However, the extent to which partial activation could benefit small-scale production units is further to be explored.

### Formulations to Deliver Pi Molecule Into the Plants

The goal of Pi fertilization to plants is to deliver Pi molecules into plant cells for their subsequent benefit functions and increase crop and benefit producers. Even applied to the soil as the commonly used method, the Pi molecule is transported from the root tissues to the leaves to enter physiological and metabolic functions. However, the large size of Pi molecules in addition to their low mobility in soil reduces their rapid absorption and uptake by roots. Foliar Pi or nano-Pi formulations could complement soil-based application and provide directly to the plant leaves the Pi, and other micronutrients, increase the efficiency of fertilizer application and enhance crop yields even under unfavorable soil conditions (Jemo et al., 2015; Avellan et al., 2018). The Pi-based nano fertilizers devoted to foliar application offer advantages of delving a relatively small quantity of Pi, bypass root uptake, and translocation mechanism steps that are energy consumed for the plants. Nanoparticles below 20–30 nm in size possess an excess of energy at the surface and are thermodynamically unstable (Avellan et al., 2018). Foliar Pi also offers the advantages of using a reduced concentration to spray at the plant leaves surface and is economically affordable for small farmers. However, the efficiency of foliar application is variable due to the interactions between the plant, the fertilizer formulation, and the environment, and achieving consistent yield responses to foliar-applied P has been elusive (Fernández et al., 2014). Foliar Pi fertilizer formulations to enhance phosphorus nutrition and biomass production deserve greater research attention (Jemo et al., 2015). Nanoscale fertilizer research and applications in agriculture are still in their infancy. A size-dependent synthesized nano-Pi to target foliar uptake processes *via* the stomata and epidermal structures as trichomes and lenticels are necessary to bypass or complement soil application methods. The nano-Pi offers advantages of resources preservation and efficiency while rapidly increase the Pi use efficiency. Promising results from soybean plants treated with nanosized hydroxyapatite showed significantly higher growth and yield of soybean plants than the conventionally P fertilizer application (Liu and Lal, 2015).

## Conclusion and Future Direction

In this study, we targeted through the present review to elucidate the role of Pi molecules in plant growth, abiotic stress alleviation, and possible Pi-dependent regulation. In this context, we first aggregated data from several studies to compare photosynthetic traits under-Pi-sufficient and poorly supplemented plants of soybeans, cowpeas, wheat, and maize as widely studied crops and common among several countries. We further respected the observations of these studies regarding shoot and root growth, shoot and root Pi concentrations under both P treatments. We then examined the biomass and P concentrations of phosphate sufficient and deficient shoots and roots under conditions of drought or heavy metal accumulation. Some of the possible novels discovered genes underlining the Pi-dependent abiotic stress regulation mechanism are also unveiled. As we assessed agronomic Pi-based intervention that could be implemented together with genetic improvements to improve crop yield at the on-farm levels. Outcomes exhibited that the rate of photosynthesis and stomatal conductance decreased under Pi starvation than in plants well treated with Pi. Conversely, the total chlorophyll contents increased in P-deficient plants, due to the lack of Pi molecules to support the functioning of photosynthesis, in particular the Rubisco function and the highly Pi-dependent fructose-1,6-bisphosphatase. Dry biomass, as well as the concentration of P in shoots and roots, were significantly reduced in Pi-deficient plants compared with well-treated plants, with marketable effects in cereals than legumes.

Under water deprivation conditions, plants with Pi supplementation exhibited higher shoot biomass than in plants without P. The concentrations of Pi in shoots and roots significantly decreased under treatments without Pi for soybean only, nevertheless, they were different between the two P treatments for the other crops. Likewise, supplementation of Pi to plants grown under heavy metal stress reduced the metal concentrations in plant leaves for Cd and Pb but could not prevent plants from absorbing the metal in the soil. We speculate that plants activated alternative pathways such as Pi-dependent glycolysis and regulation of mitochondrial respiration in the cytoplasm to alleviate drought stress. Pi molecules down-regulate the synthesis of phytohormones, abscisic acid, indoleacetic acid, and reactive oxygen compounds. To detoxify themselves from heavy metal stress, plants enhance the activity of catalase and ascorbate peroxidase which prevents lipid peroxidation in the leaves and reduces metal in the cell. Among the different genes evaluated, the family of *HvPIP* genes is overexpressed under combined the Pi and drought stresses, suggesting a probable control of water movement during the two stresses. The *PHO1* gene is expressed as well to improve survival of plants under Pi stress and to regulate the transduction of the ABA signal under salt stress.

Various agronomic Pi interventions to increase Pi at the farm level were reviewed highlighting the pros and cons of each intervention. Conventional water-soluble fertilizer has only 20% agronomic efficiency due to its rapid solubility. While the partial activation of PR offers a promising avenue for the development of slow/controlled release fertilization. Microbial technology could play an important role in organic farming, but the amount of nutrients released is still unpredictable. Likewise, the formulation at the nanoscale and the method of foliar fertilization if successful can contribute to greater agronomic efficiency and conservation of resources. Possible limitations of the study could be a broad approach to circumvent the roles of Pi in plant growth and abiotic stress alleviation. In light of outcomes, we considered designing a review work with a complete picture of the Pi mechanism of the plant when developing our review hypothesis. Another point was the role of Pi molecules in the homeostasis of legume nodules that we could not cover in the present work, but it will be studied in another review article. Overall, the review helps improve our understanding of the progress made in the integrated development of Pi interventions to improve agricultural production under conditions of abiotic stress.

## Author Contributions

MJ: coordinated the review. NB: drafted the first version of the manuscript. NB, MR, and MJ: retrieved the data. NB and MJ: curated and analyzed the data. NB, MR, AR, KO, MH, and MJ: wrote and revised the whole manuscript. MR: constructed the database used in the accepted version of the manuscript. MH: reviewed the manuscript and provide guidance. All authors contributed to the article and approved the submitted version.

## Conflict of Interest

The authors declare that the research was conducted in the absence of any commercial or financial relationships that could be construed as a potential conflict of interest.

## Publisher's Note

All claims expressed in this article are solely those of the authors and do not necessarily represent those of their affiliated organizations, or those of the publisher, the editors and the reviewers. Any product that may be evaluated in this article, or claim that may be made by its manufacturer, is not guaranteed or endorsed by the publisher.

## Abbreviations

3-PGA: 3 -phosphoglycerate
ABA: abscisic acid
ATP: Adenosine triphosphate
IAA: indoleacetic acid
NADPH: Nicotinamide adenine dinucleotide phosphate
P: phosphorus
PR: Phosphate rock
Pi: inorganic phosphate
Po: organic phosphate
ROS: reactive oxygen species
RuBisCO, Ribulose-1: 5-bisphosphate carboxylase-oxygenase
RuBP: ribulose-1-5-biphosphate
SOD: superoxide dismutase.
