# Supplementary material for: Phosphate-Dependent Regulation of Growth and Stresses Management in Plants
Source: Front Plant Sci. 2021 Oct 28;12:679916. doi: 10.3389/fpls.2021.679916 (PMC8581177; doi:10.3389/fpls.2021.679916)
Supplement: Supplementary file 1 [file Table_1.pdf]

Phosphate-dependent regulation of growth and stresses management in plants

N. Bechtaoui[1, *], Muhammad Rabiu Kabiru [1,2]; A. Raklami[3], K. Oufdou[3,1], Mohamed Hafidi[3,1] and M. Jemo[1, *]

[1] AgroBiosciences Program, University Mohammed VI Polytechnic (UM6P), Benguerir, Morocco.
[2] Centre for Dryland Agriculture, Bayero University, P.M.B. 3011, Kano, Nigeria
[3] Laboratory of Microbial Biotechnology, Agrosciences, and Environment (BioMAgE), Faculty of Sciences Semlalia, Cadi Ayyad University, Marrakech, Morocco

* Author for correspondences contacts (Martin Jemo and Noura Bechtaoui (e-mails: martin.jemo@um6p.ma and noura.bechtaoui@um6p.ma)

Supplementary Table

Table S1. Photosynthesis, growth and uptake, and heavy metals stress, sources of phosphorus, species, and references from which data were retrieved for the meta-analysis.

	Phosphorus source	Plant traits	*Species*	References
Photosynthesis	KH_2PO_4	Net photosynthesis, Shoot dry weight, stomatal conductance, total chlorophyll,	*Glycine max, Vigna unguiculata (L.), Triticum aestivum, Triticum aestivum, Zea mays*	Chu et al., 2018 ; Moreira et al., 2017 ; Bulgarelli et al., 2017 ; Zhu et al., 2020 ; Izaguirre-Mayoral et al., 2002 ; Meena et al., 2021 ; Taffouo et al., 2014; Sherchand and Paulsen, 1985; Shabbir et al., 2015; Zhengping et al., 2004; Jia et al., 2018; Wang et al., 2017
	NH4H2PO4		*Triticum aestivum*	Rabbi et al., 2017; Arshad et al., 2015
	$CaH_4O8P_2H_2O$		*Triticum aestivum , Zea mays*	Ozturk et al., 2005; Wang and Ning, 2019
	NPK		*Triticum aestivum*	Choudhary and Kumar, 2014; Beinsan et al., 2020
	TSP		*Vigna unguiculata (L.), Triticum aestivum*	Fouda and Abd-Elhamied, 2017; Nyoki and Ndakidemi, 2014; Nursu'aidah et al., 2014; Moreira et al., 2020; El-Sobky, 2017; Jemo et al., 2017.
	RP		*Zea mays*	Aliet al., 2014
Growth and uptake	KH_2PO_4	Shoot dry weight, shoot P concentration, Root P concentration	*Glycine max, Vigna unguiculata (L.), Zea max*	Jin et al., 2006; Chiulele, 2003; Nafady and Elgharably, 2018; Tang et al., 2019
	$NH_4H_2PO_4$		*Glycin max*	He et al., 2019; Feng et al.,2021; He et al.,2017; He et al.,2021
	$CaH_4O8P_2H_2O$		*Glycin max, Triticum aesticum, Zea mays*	Rotaru and Risnoveanu, 2019; Yu et al., 2016; Zhan et al., 2019
	SSP			Lima et al., 2017; Rajkhowa, 2017
	TSP			Karimzadeh et al., 2021; HI Mohammadi et al.,2020; Kaya et al., 2020; Jemo et al., 2017.
Heavy metals stress	KH_2PO_4	Shoot-Cd, -Pb -Zn concentrations	*Sedum alfredii,Triticum aestivum, Zea mays*	Huang et al., 2012; Zhang et al., 2016
	$NH_4H_2PO_4$		*Oryza sativa L; Triticum aestivum*	Qayyum et al., 2017; Rehman et al., 2018
	SSP			Elgharably, 2020; Lavado et al., 2001; Neu et al., 2020; Bashir et al., 2018; Nafady and Elgharably, 2018
	TSP		*Brassica napus L, Hordeum vulgare L, Zea mays, Triticum aestivum*	Lavado et al., 2001; Neu et al., 2020,
	Activated RP		*Oryza sativa L; Triticum aestivum*	Ahmad et al., 2019; Bashir et al., 2018; Elgharably, 2020
